# Insulin resistance, kidney outcomes and effects of the endothelin receptor antagonist atrasentan in patients with type 2 diabetes and chronic kidney disease

**DOI:** 10.1186/s12933-023-01964-8

**Published:** 2023-09-16

**Authors:** J. David Smeijer, Donald E. Kohan, Peter Rossing, Ricardo Correa-Rotter, Adrian Liew, Sydney C.W. Tang, Dick de Zeeuw, Ron T. Gansevoort, Wenjun Ju, Hiddo J. Lambers Heerspink

**Affiliations:** 1 Department of Clinical Pharmacy and Pharmacology, University Medical Center Groningen, University of Groningen, Groningen, The Netherlands; 2 Division of Nephrology, University of Utah Health, Salt Lake City, UT USA; 3 Steno Diabetes Center Copenhagen, Herlev, Denmark; 4 Department of Clinical Medicine, University of Copenhagen, Copenhagen, Denmark; 5 National Medical Science and Nutrition Institute Salvador Zubirán, Mexico City, Mexico; 6 Mount Elizabeth Novena Hospital, Singapore, Singapore; 7 George Institute for Global Health, Newtown, Australia; 8 Division of Nephrology, Department of Medicine, The University of Hong Kong, Hong Kong, Hong Kong; 9 Department of Internal Medicine, University Medical Center Groningen, University of Groningen, Groningen, The Netherlands; 10 Department of Computational Medicine and Bioinformatics, University of Michigan Medical School, Ann Arbor, MI USA

**Keywords:** Insulin resistance, Atrasentan, Endothelin receptor antagonist, Chronic kidney disease, Type 2 diabetes

## Abstract

**Background:**

Insulin resistance (IR) is a pathophysiologic hallmark of type 2 diabetes and associated with the presence of chronic kidney disease (CKD). Experimental studies suggest that endothelin-1 increases IR. We assessed the association between IR and cardio-renal outcomes and the effect of the selective endothelin receptor antagonist atrasentan on IR in patients with type 2 diabetes and CKD.

**Methods:**

We used data from the RADAR and SONAR trials that recruited participants with type 2 diabetes and CKD [eGFR 25–75 mL/min/1.73 m², urine albumin-to-creatinine ratio of 300–5000 mg/g]. IR was calculated using the homeostatic model assessment (HOMA-IR). The association between HOMA-IR and the pre-specified cardio-renal outcomes was assessed using multivariable Cox proportional hazards regression, and effects of atrasentan on HOMA-IR by a linear mixed effect model.

**Results:**

In the SONAR trial, each log-unit increase in HOMA-IR was associated with an increased risk of the composite cardio-renal outcome [hazard ratio 1.32 (95%CI 1.09,1.60; p = 0.004)], kidney outcome [hazard ratio 1.30 (95%CI 1.00,1.68; p-value = 0.048)], and the kidney or all-cause mortality outcome [hazard ratio 1.25 (95%CI 1.01,1.55; p-value = 0.037)]. After 12 weeks treatment in the RADAR trial (N = 123), atrasentan 0.75 mg/day and 1.25 mg/day compared to placebo reduced HOMA-IR by 19.1 (95%CI -17.4, 44.3) and 26.7% (95%CI -6.4, 49.5), respectively. In the SONAR trial (N = 1914), atrasentan 0.75 mg/day compared to placebo reduced HOMA-IR by 9.6% (95%CI 0.6, 17.9).

**Conclusions:**

More severe IR is associated with increased risk of cardio-renal outcomes. The endothelin receptor antagonist atrasentan reduced IR.

**Trial registration:**

RADAR trial (Reducing Residual Albuminuria in Subjects With Diabetes and Nephropathy With AtRasentan): NCT01356849.

SONAR trial (The Study Of Diabetic Nephropathy With AtRasentan) NCT01858532.

**Supplementary Information:**

The online version contains supplementary material available at 10.1186/s12933-023-01964-8.

## Background

Insulin resistance (IR) is a pathophysiological hallmark of type 2 diabetes with reduced insulin sensitivity being detectable up to 5 years prior to the diagnosis of type 2 diabetes [1].

The pathophysiological processes associated with IR can lead to kidney impairment prior to the diagnosis of type 2 diabetes, as increased IR is associated with both the presence of microalbuminuria and chronic kidney disease (CKD) in subjects without diabetes [2–4]. Moreover, in the general population and in newly diagnosed patients with type 2 diabetes, a higher IR is independently associated with a faster rate of glomerular filtration rate decline [5–7].

Binding of endothelin-1 (ET-1) to the endothelin A (ET_A_) receptor induces glomerular/tubular dysfunction, inflammation, and fibrosis in both diabetic and non-diabetic kidneys [8]. There appears to be a reciprocal association between ET-1 and IR resulting in a vicious circle of increased organ damage. In vitro research demonstrated that intracellular pathways involved in insulin-mediated glucose uptake in adipocytes and vascular smooth muscles cells are disrupted by ET-1 [9, 10]. Conversely, IR increases renal ET-1 levels and ET_A_ receptor expression [11, 12]. In clinical studies, IR increased in response to administration of exogenous ET-1 in healthy volunteers supporting the close relation between ET-1 and IR [13].

Selective endothelin receptor A antagonists (ERA) have been shown to slow the progression of CKD. Low dose atrasentan, an ERA, decreased albuminuria and reduced the risk of major kidney outcomes in patients with type 2 diabetes and CKD [14, 15]. A small study demonstrated that administration of an ERA reduces IR in obese patients with coronary artery disease [16]. In addition, a small observational study reported a significant reduction in HbA1c in patients with pulmonary hypertension using ERA [17]. In the RADAR dose-finding study, the ERA atrasentan reduced HbA1c compared to placebo in patients with type 2 diabetes and CKD possibly mediated by improvements in IR [18]. However, because of the small sample size and short follow-up, these results are prone to chance findings and can only be interpreted as hypothesis generating. Larger studies with longer follow-up are thus required to provide more robust evidence about the magnitude and time-course of the effects of ERA treatment on IR and glycemic control.

Therefore, the aims of this study were firstly to assess the association between baseline IR and clinical outcomes in a large well characterized cohort of patients already diagnosed with type 2 diabetes and CKD. Secondly, we assessed whether the ERA atrasentan reduces IR and HbA1c in patients with type 2 diabetes and CKD.

## Methods

### Study design and patient population

This study is a post-hoc analysis of the RADAR and SONAR clinical trials. The study design and primary results of both trials have been published before [14, 15, 19, 20]. In short, the Reducing Residual Albuminuria in Subjects With Diabetes and Nephropathy With AtRasentan (RADAR) trial was a randomized double-blind phase 2b clinical trial recruiting 153 patients with type 2 diabetes, urinary albumin-to-creatinine ratio [UACR] ≥ 300 - ≤3500 mg/g, and an estimated glomerular filtration rate (eGFR) of ≥ 30 - ≤75 mL/min/1.73m^2^ receiving maximum tolerated labeled dose of renin-angiotensin-system (RAS) inhibitors. Patients were randomly assigned to 12 weeks treatment with atrasentan 0.75 mg/day, atrasentan 1.25/mg day or matched placebo. The primary efficacy endpoint of the trial was the change in UACR from baseline to week 12 [18].

The Study Of Diabetic Nephropathy With AtRasentan (SONAR) trial recruited patients between 18 and 85 years old with type 2 diabetes, UACR ≥ 300 - < 5000 mg/g, and eGFR of ≥ 25 - < 75 mL/min per 1.73 m [2]. After screening and run-in to optimize RAS inhibitor treatment, eligible participants proceeded to a 6-week open-label enrichment period, during which all patients received 0.75 mg/day atrasentan. Atrasentan responders were defined as patients with ≥ 30% reduction in UACR who did not have substantial fluid retention (defined as an increase in body weight of ≥ 3 kg and a B-type natriuretic peptide (BNP) increase ≥ 300 pg/mL), and who did not have an increase in serum creatinine of more than 0·5 mg/dL and 20% from baseline. After six weeks enrichment, 2648 responders and 1020 non-responders without overt signs of fluid retention were randomly assigned to continue 0.75 mg/day atrasentan or to transition to placebo [15, 20]. The RADAR and SONAR trials were designed and conducted in accordance with national regulatory and ethical guidelines and are registered with ClinicalTrials.gov (NCT01356849 and NCT01858532).

### Insulin resistance

Insulin resistance was calculated using the homeostatic model assessment (HOMA-IR): [(fasting plasma insulin [FPI] × fasting plasma glucose [FPG])/22.5] [21]. Blood samples for measurement of insulin and glucose were taken while patients were in a fasted state. Insulin and glucose were measured at the start and end of the 6-week enrichment period, randomization, at 1 and 12 months post-randomization, and annually thereafter. In RADAR, HOMA-IR was determined in 123 patients (80.4% of the overall cohort). In SONAR, 1102 patients were available for analysis of the association between HOMA-IR at baseline of the enrichment phase and long-term outcomes. At randomization, HOMA-IR was assessed in 1914 patients. In this subgroup, the effect of atrasentan versus placebo was assessed on HOMA-IR and HbA1c over time.

### Endpoints

We investigated four cardio-renal clinical endpoints: (1) the composite cardiovascular (CV)-kidney endpoint defined as time from randomization to first occurrence of a sustained doubling of serum creatinine, end-stage kidney disease ([ESKD] defined as eGFR < 15 mL/min/1.73 m [2], need for chronic dialysis, renal transplantation), CV death, non-fatal myocardial infarction (MI) or non-fatal stroke; [20] (2) the composite kidney or all-cause mortality endpoint defined as time from randomization to first occurrence of doubling of serum creatinine from baseline (confirmed by 30-day serum creatinine), ESKD or death; (3) the composite kidney endpoint which was defined as time to doubling of serum creatinine from baseline (confirmed by 30-day serum creatinine) or ESKD; and (4) the CV composite endpoint defined as time to CV death, non-fatal MI and non-fatal stroke.

### Statistics

Summary statistics were used to describe the demographic and clinical characteristics of patients included in the IR analysis and the randomized RADAR and SONAR trial populations. We log-transformed HOMA-IR, UACR and brain natriuretic peptide (BNP) values before analysis to take into account their skewed distribution. Cox proportional hazards regression was used to assess the association between baseline HOMA-IR and the relative hazard of the CV-kidney outcomes. In addition, HOMA-IR in tertiles were categorized and estimated the hazard ratio using the lower tertile as a common reference for the middle and upper tertiles. Three regression models were used: (1) a baseline model with treatment, age and sex as covariates; (2) the baseline model adding eGFR, log(UACR), body mass index (BMI) and systolic- and diastolic blood pressure as additional covariates; and (3) the final model that included all aforementioned covariates plus hemoglobin, insulin use, CV disease history and BNP. All covariates were measured at baseline of the enrichment phase before patients were exposed to atrasentan.

We used the RADAR and SONAR trials for determination of the effect of atrasentan on IR. Analysis of co-variance was used in RADAR to estimate the effect of atrasentan 0.75 mg/day and 1.25 mg/day compared to placebo on HOMA-IR. An unpaired t-test was performed to compare the natural logarithm of HOMA-IR between start of the open-label enrichment phase and the randomization visit. A linear mixed effect model was used to assess the effect of atrasentan compared to placebo on changes in HOMA-IR from randomization. The model included treatment, visit and interactions between treatment and visit as categorical fixed effects. An unstructured variance–covariance matrix was used to allow for correlations and general patterns of standard deviations across the repeated outcome measurements. Linear mixed models were used to assess whether the effect of atrasentan compared to placebo on IR was consistent across subgroups by baseline age, sex, eGFR, UACR, BMI and insulin by including a fixed effect for the subgroup and three-way interaction between treatment, visit and subgroup. All analysis were performed with the software package ‘R’, version 4.2.0. (R Foundation for Statistical Computing, Vienna, Austria).

## Results

### Patient disposition and baseline characteristics

Baseline characteristics of RADAR participants with available HOMA-IR data (N = 123) are described in Additional file 1: Table S1. Out of 3668 patients randomized in the SONAR trial, 1102 (30.0%) were included in the analysis of the association between baseline IR and long-term cardio-renal outcomes. Reasons for exclusion were unavailable insulin measurement at baseline of enrichment (n = 2329), insulin measurements taken while not fasting (n = 186) and missing information on other covariates (n = 51). Across progressively higher IR tertiles, patients in the highest tertile had a higher BMI, higher hemoglobin and hematocrit at baseline, were more likely to be of female sex, and more like to use insulin and statins (Table 1). Baseline characteristics were generally representative of the overall SONAR trial population (Additional file 2: Table S2).

**Table 1:** Demographic and clinical characteristics at the start of the enrichment phase according to tertiles of Insulin Resistance

Characteristics	Tertile 1 (n = 366)	Tertile 2 (n = 369)	Tertile 3 (n = 367)	p-value for trend
HOMA-IR	2.3 [0.0-3.9]	5.9 [3.9–10.3]	19.6 [10.4–286.0]	NA
Age, years	63.1 (9.4)	64.4 (8.6)	63.6 (8.2)	0.445
Sex, n (%)				
Women	72 (19.7%)	114 (30.9%)	99 (27.0%)	**0.002**
Men	294 (80.3%)	255 (69.1%)	268 (73.0%)	
Race, n (%)				
Asian	166 (45.4%)	139 (37.7%)	108 (29.4%)	**< 0.001**
Black	13 (3.6%)	29 (7.9%)	21 (5.7%)	
Other	27 (7.4%)	12 (3.3%)	12 (3.3%)	
White	160 (43.7%)	189 (51.2%)	226 (61.6%)	
Body weight (kg)	76.2 (15.5)	83.3 (18.0)	92.0 (21.7)	**< 0.001**
BMI, kg/m^2^	27.6 (4.6)	30.2 (5.6)	32.7 (6.3)	**< 0.001**
Blood pressure (mmHg)				
Systolic	136.6 (16.2)	137.5 (16.2)	137.7 (14.6)	0.338
Diastolic	75.6 (9.7)	75.5 (10.3)	76.6 (9.3)	0.162
eGFR, ml/min 1.73m2	42.4 (12.9)	41.2 (12.0)	41.4 (12.2)	0.311
UACR, mg/g	888 [514–1773]	815 [469–1657]	881 [452–1570]	0.258
Haemoglobin, g/L	125.9 (17.3)	128.8 (17.0)	131.2 (17.5)	**< 0.001**
BNP, pg/mL	51 [27–95]	46 [23–87]	44 [26–86]	0.294
Hematocrit, L/L	0.38 (0.05)	0.39 (0.05)	0.40 (0.05)	**< 0.001**
CVD history, n (%)	75 (18.8%)	82 (21.9%)	84 (22.2%)	0.308
Insulin use, n (%)	136 (37.2%)	260 (70.5%)	304 (82.8%)	**< 0.001**
Diuretic use, n (%)	282 (77.0%)	309 (83.7%)	290 (79.0%)	0.066
Statin use, n (%)	272 (74.3%)	299 (81.0%)	301 (82.0%)	**0.020**

**Abbreviations:** HOMA-IR = Homeostatic Model Assessment for Insulin Resistance; BMI = body mass index; eGFR = estimated glomerular filtration rate; UACR = urine albumin creatinine ratio; BNP = B-type natriuretic peptide; CVD = cardiovascular disease

**Note:** HOMA-IR: median (min and max values per tertile); UACR and BNP: median (interquartile range); all other numerical values: mean (standard deviation); Regarding p-value for difference: for continues variables linear regression was performed with the variable of interest as dependent variable and IR tertile as numerical covariate to assess the presence of a significant trend across tertiles. For categorical variables a chi-square test was performed to assess the presence of a significant difference in distribution across tertiles

### Long term outcomes

During follow-up, 103, 86, 62, and 43 participants experienced a CV-kidney, kidney or all-cause mortality, kidney, and CV endpoints, respectively. Cox proportional hazard regression with adjustment for patient demographics, randomized treatment and cardiovascular risk markers, including eGFR, UACR, BNP and CV disease history showed that each log increament in HOMA IR was significantly associated with a higher risk of the CV kidney, kidney or all-cause mortality and kidney composite outcomes with corresponding HRs per log increment baseline HOMA-IR in the fully adjusted model of 1.32 (95%CI 1.09,1.60, p = 0.004); 1.25 (95%CI 1.01, 1.55, p = 0.037); 1.30 (95%CI 1.00-1.68, p = 0.048); 1.34 (95%CI 0.99, 1.81, p = 0.060); for the CV-kidney, kidney or all-cause mortality, kidney, and CV outcomes, respectively (Table 2). Repeating the analyses for participants not using insulin at baseline (n = 402) yielded similar results as our main analyses (Additional file 3: Table S3).

**Table 2:** Association between baseline HOMA-IR and long-term cardio-renal outcomes

Outcome	n/N events (%)	Model 1	*p* value	Model 2	*p* value	Model 3	*p* value
		HR (95% CI)		HR (95% CI)		HR (95% CI)	
Cardiorenal outcomes							
Tertile 1	27/339 (8.0%)	(reference)		(reference)		(reference)	
Tertile 2	37/332 (11.1%)	1.31 (0.79–2.17)	0.289	1.41 (0.84–2.39)	0.197	1.74 (1.01–3.01)	**0.046**
Tertile 3	39/328 (11.9%)	1.44 (0.88–2.35)	0.149	1.64 (0.96–2.81)	0.072	2.29 (1.28–4.09)	**0.005**
*p* value for trend across tertiles	*NA*	1.19 (0.94–1.51)	0.154	1.27 (0.98–1.65)	0.076	1.49 (1.12–1.97)	**0.006**
per log unit increase	*NA*	1.13 (0.95–1.35)	0.163	1.19 (0.99–1.43)	0.069	1.32 (1.09–1.60)	**0.004**
**Renal composite or all-cause mortality**							
Tertile 1	23/343 (6.7%)	(reference)		(reference)		(reference)	
Tertile 2	29/340 (8.5%)	1.20 (0.69–2.10)	0.511	1.25 (0.70–2.24)	0.453	1.40 (0.76–2.56)	0.278
Tertile 3	34/333 (10.2%)	1.48 (0.87–2.51)	0.149	1.61 (0.89–2.90)	0.116	2.06 (1.09–3.88)	**0.026**
*p* value for trend across tertiles	*NA*	1.22 (0.93–1.58)	0.145	1.27 (0.95–1.70)	0.110	1.44 (1.05–1.97)	**0.023**
per log unit increase	*NA*	1.12 (0.93–1.36)	0.240	1.16 (0.95–1.43)	0.151	1.25 (1.01–1.55)	**0.037**
**Renal composite**							
Tertile 1	16/350 (4.6%)	(reference)		(reference)		(reference)	
Tertile 2	23/346 (6.6%)	1.38 (0.72–2.64)	0.331	1.46 (0.72–2.93)	0.293	1.87 (0.90–3.88)	0.093
Tertile 3	23/344 (6.7%)	1.48 (0.78–2.81)	0.235	1.51 (0.71–3.18)	0.281	2.49 (1.11–5.60)	**0.028**
*p* value for trend across tertiles	*NA*	1.20 (0.88–1.64)	0.244	1.21 (0.84–1.74)	0.305	1.55 (1.05–2.30)	**0.029**
per log unit increase	*NA*	1.09 (0.87–1.37)	0.443	1.11 (0.87–1.43)	0.406	1.30 (1.00-1.68)	**0.048**
**CV composite**							
Tertile 1	12/354 (3.4%)	(reference)		(reference)		(reference)	
Tertile 2	15/354 (4.2%)	1.22 (0.57–2.62)	0.609	1.42 (0.65–3.11)	0.385	1.61 (0.71–3.66)	0.258
Tertile 3	16/351 (4.6%)	1.34 (0.63–2.84)	0.445	1.61 (0.72–3.62)	0.249	1.88 (0.78–4.51)	0.160
*p* value for trend across tertiles	*NA*	1.15 (0.80–1.67)	0.448	1.26 (0.85–1.88)	0.253	1.35 (0.88–2.07)	0.167
per log unit increase	*NA*	1.17 (0.89–1.52)	0.256	1.25 (0.95–1.66)	0.116	1.34 (0.99–1.81)	0.060

**Note**:

Model 1 covariates: age, sex, treatment assignment (Atrasentan or Placebo)

Model 2 covariates: age, sex, treatment assignment (Atrasentan or Placebo), race, BMI, eGFR, log(UACR), SBP, DBP

Model 3 covariates: age, sex, treatment assignment (Atrasentan or Placebo), race, BMI, eGFR, log(UACR), SBP, DBP, hemoglobin, insulin use, cardiovascular disease history, log(BNP)

### Effect of atrasentan on insulin resistance

**Fig. 1:**
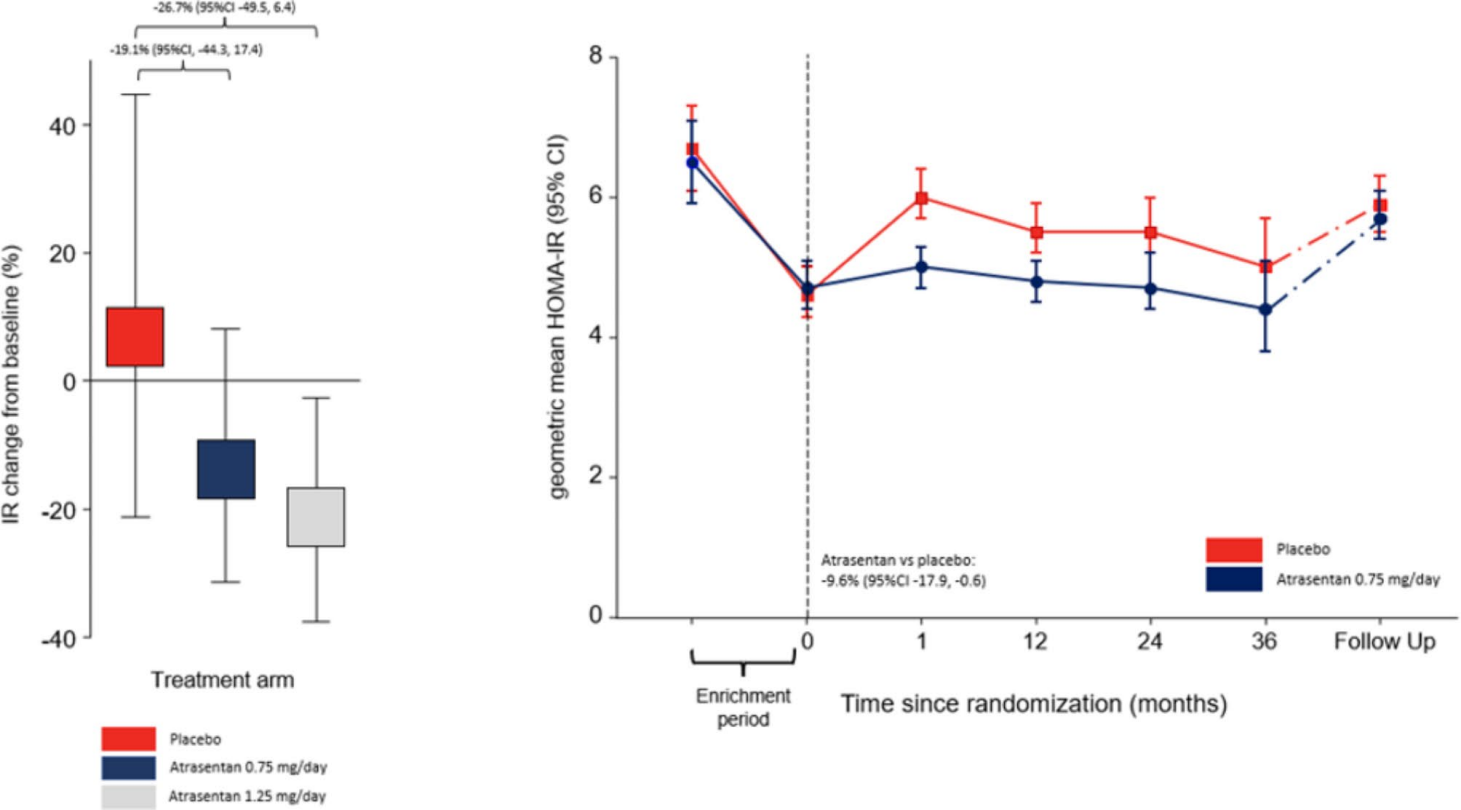
Atrasentan reduces insulin resistance. *Panel A*: Change from baseline in HOMA-IR in the RADAR trial. *Panel B*: Geometric mean HOMA-IR values per study visit in the SONAR trial. During the enrichment period of the SONAR trial all patients received atrasentan 0.75 mg/day. Enrichment results of patients subsequently randomized to continue atrasentan or to transition to placebo are presented separately

**Fig. 2:**
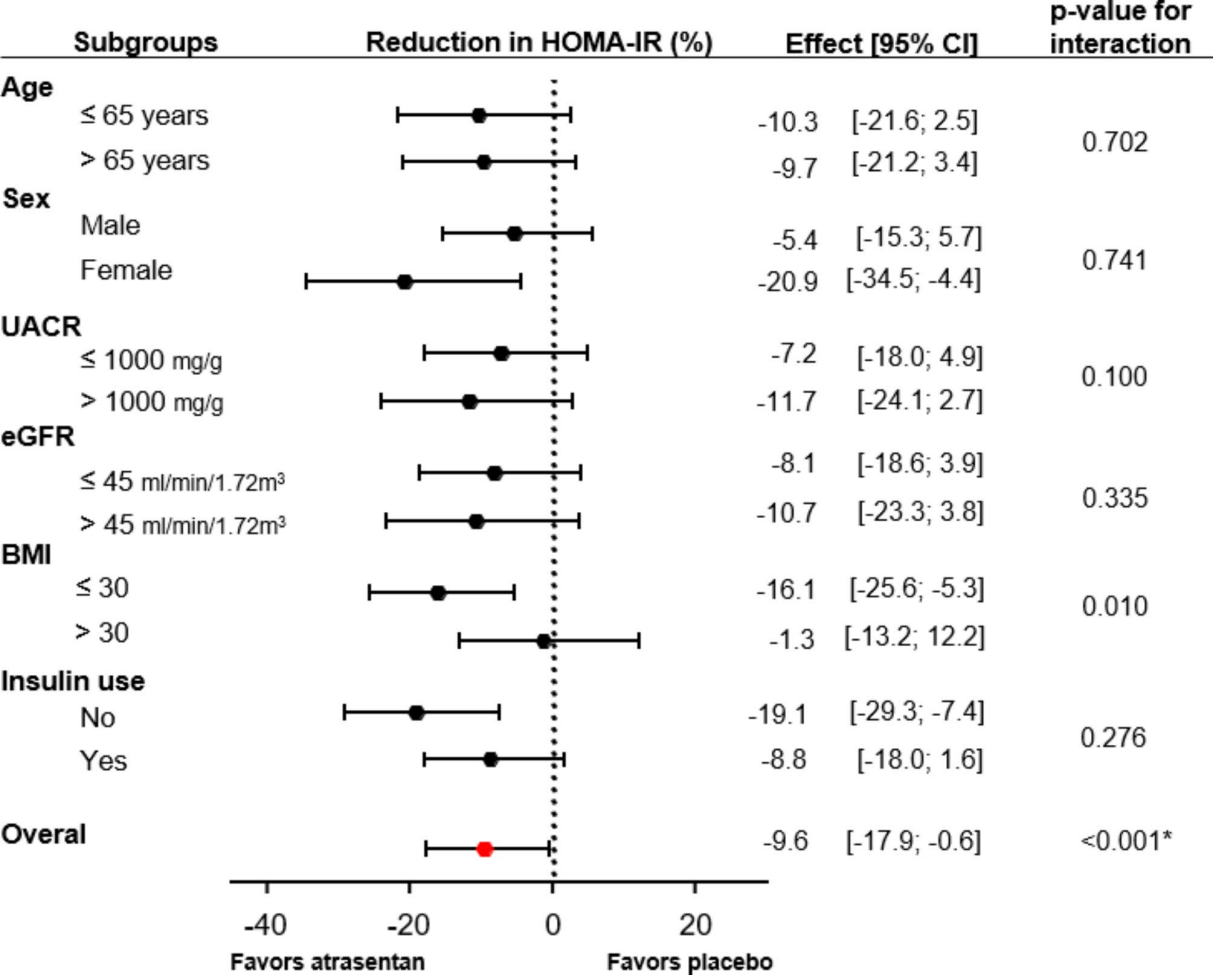
Treatment effect of atrasentan on HOMA-IR during the SONAR double-blind phase. Note: * p-value for atrasentan versus placebo for all patients combined averaged over visits

In the placebo group (N = 26) of the RADAR trial, HOMA-IR slightly increased by 7.8% (95% CI -20.3, 45.7) after 12 weeks follow-up, while HOMA-IR decreased in patients randomized to atrasentan 0.75 mg/day (N = 47) and 1.25 mg/day (N = 50) by -12.9% (95%CI -30.5, 9.2) and − 21.0% (95%CI -36.5, -1.7%), respectively, resulting in a difference with placebo of -19.1% (95%CI -44.3, 17.4; p = 0.266) and − 26.7% (95%CI -49.5, 6.4; p = 0.102), respectively (Fig. 1A). In the SONAR trial, geometric mean HOMA-IR at the start of the 6-week enrichment period was 6.8 units (95%CI 6.4, 7.1) which decreased to 4.7 units (95%CI 4.4, 4.9; p vs. baseline < 0.0001) after 6-weeks treatment with 0.75 mg/day atrasentan. One month after randomization, HOMA-IR increased in the placebo group from 4.6 units (95%CI 4.3, 5.0) to 5.5 units (95%CI 5.1, 5.9, p < 0.001), corresponding to a 21.5% (95%CI 16.5, 26.9) increase. HOMA-IR remained at 4.8 units (95%CI 4.4–5.2) in the atrasentan group corresponding to a 2.3% (95%CI -1.7, 7.0) change (Fig. 1B). During the remainder of the double-blind treatment period, patients randomized to atrasentan had a significantly lower HOMA-IR compared to patients on placebo resulting in a between-group difference of -9.6% [95%CI -17.9, -0.6, p < 0.001]) (Fig. 1B). Six weeks following study drug discontinuation, HOMA-IR levels were similar between the placebo and atrasentan group (Fig. 1B). The effect of atrasentan compared to placebo was consistent in subgroup analyses by age, sex, baseline UACR, baseline eGFR and use of insulin (Fig. 2). Among patients with baseline BMI < 30 kg/m^2^ the effect of atrasentan was more pronounced compared to those with a BMI > 30 kg/m^2^ (p for interaction 0.010; Fig. 2). During the double-blind phase, atrasentan treated patients had a significantly lower HbA1c compared to placebo treated patients resulting in a between-group difference in HbA1c of 0.18% (95%CI 0.05,0.31, p-value < 0.001) (Additional file 4: Supplementary Fig. 1).

## Discussion

In patients with type 2 diabetes and CKD at high risk of kidney failures and CV events, more severe IR was independently associated with a higher risk of kidney and CV outcomes. In these high-risk patients, low dose atrasentan reduced IR by approximately 10% with consistent effects irrespective of baseline kidney function or IR.

To our knowledge, this is the first prospective study that demonstrates that higher IR is independently associated with a higher risk of cardio-renal outcomes in patients with type 2 diabetes mellitus and CKD. Previous cross-sectional studies already demonstrated an association between IR and kidney disease, but there are very few prospective studies [2–4]. Zhang et al. found that among subjects with normal glucose tolerance, those in the highest HOMA-IR quartile had a 50% increased risk of developing microalbuminuria [5]. In the general population, an increase in HOMA-IR during a 6 year baseline period was associated with an increased risk of adverse kidney outcomes during subsequent 6 years follow-up (n = 5347) [7]. However, these studies did not include patients with type 2 diabetes and CKD. We found a higher level of IR to be associated with an increased risk of decreased kidney function in a well-characterized international cohort of patients with type 2 diabetes and CKD. Our findings support a prior study on a new classification of diabetes which demonstrated that newly diagnosed patients with type 2 diabetes and preserved kidney function and characterized by severe insulin resistance had a higher risk of developing CKD compared to those not characterized by high insulin resistance [6]. Both cardiovascular events as well as kidney events contributed to the increased risk of developing the cardiovascular composite outcome, a prespecified adjudicated outcome in the SONAR trial. Our findings suggest that even after development of overt nephropathy, the presence of IR is associated with a higher risk of adverse clinical outcomes.

Using both the RADAR and SONAR trials allowed for a comprehensive characterization of the effect of atrasentan on HOMA-IR as the trials complemented each other in various ways. The RADAR trial was a relatively small dose-finding trial with a placebo comparison to generate the hypothesis that atrasentan reduces IR. The small sample size of the RADAR may have resulted in an overestimation of the effect size [22, 23]. The results of the larger SONAR confirm the beneficial effect of atrasentan on IR with a more robust and precise effect estimate. Additionally, while IR reduced in the atrasentan group after a relatively short follow-up period of 12 weeks in RADAR, SONAR confirmed that these initial effects are sustained over more than 2 years of follow-up. Moreover, SONAR demonstrated that the reduction in IR is reversible among participants who switched from atrasentan to placebo at randomization further supporting a true pharmacologic effect. Finally, HbA1c also reduced with atrasentan in the SONAR trial as previously observed in RADAR [18].

The effects of atrasentan on IR were consistent in most examined subgroups. It is not entirely clear why the effect of atrasentan on IR was more pronounced among participants with a lower BMI. It is possible that the higher percentage of patients on insulin in the high BMI group may influence the HOMA-IR measurement although in a subgroup analysis the effect of atrasentan was not different in those using and non-using insulin. It could also be possible that pharmacokinetic effects of atrasentan have played a role since a prior study reported an association between higher body weight (and BMI) and lower atrasentan exposure [24].

The key strength of this analysis was that the data were derived from two randomized controlled trials which were conducted to high standards and enrolled a broad internationally representative patient cohort with type 2 diabetes and CKD. The multiple IR measurements over time allowed for a comprehensive evaluation of the effect of atrasentan. This study also has limitations, the most obvious being that insulin measurements were only available for a subset of SONAR participants. In addition, although HOMA-IR is a widely used feasible method for the assessment of insulin resistance, considerable random variability in HOMA-IR levels exists [25, 26]. Although some studies reported that HOMA-IR provides a valid estimate of insulin sensitivity in patients with type 2 diabetes, other studies reported that HOMA-IR may not be a reliable predictor of insulin resistance compared to the ‘gold standard’ euglycemic clamp technique in certain populations such as older patients with poorly controlled diabetes [25, 27, 28]. Also, the kidneys play an important role in the clearance of insulin and impaired kidney function could have influenced insulin levels in addition to underlying metabolic disturbances related to type 2 diabetes. Insulin treatment may have affected the IR assessments as well, but all blood samples were taken in fasted states during which we would not expect patients to use short acting insulin. The model adjusted for use of insulin, in addition we performed a separate analysis of patients not treated with insulin, which did not influence the findings. Finally, because this is a post-hoc analysis, we cannot exclude chance findings.

## Conclusions

In conclusion, IR is associated with a higher risk of developing cardio-kidney outcomes in patients with type 2 diabetes and CKD. The ERA atrasentan reduces IR, a finding which was consistent across most patient subgroups. Whether the reduction in IR contributes to the long-term kidney protective effects of atrasentan requires further study.

### Electronic supplementary material

Below is the link to the electronic supplementary material.

Additional File 1: Table S1 with patient characteristics of the RADAR trial.

Additional File 2: Table S2 with baseline characteristics of SONAR trial population for long-term outcomes.

Additional File 3: Table S3 with Association between HOMA-IR and long-term cardio-renal outcomes in participants not using insulin.

Additional File 4: Figure S1 with HbA1c during the SONAR trial for patients with a HbA1c measurement at randomization.

## Data Availability

These clinical trial data can be requested by any qualified researchers who engage in rigorous, independent scientific research and will be provided after review and approval of a research proposal and statistical analysis plan and execution of a data sharing agreement by the corresponding author and the SONAR Steering Committee. Data requests can be submitted at any time.

## Abbreviations

BMI: Body mass index
BNP: B-type natriuretic peptide
CKD: Chronic kidney disease
CV: Cardiovascular
eGFR: Estimated glomerular filtration rate
ERA: Endothelin receptor A antagonists
ESKD: End-stage kidney disease
ET-1: Endothelin-1
ET_A_: Endothelin A receptor
FPG: Fasting plasma glucose
FPI: Fasting plasma insulin
HOMA-IR: Homeostatic model assessment
IR: Insulin resistance
MI: Myocardial infarction
RADAR: Reducing Residual Albuminuria in Subjects With Diabetes and Nephropathy With AtRasentan
RAS: Renin-angiotensin-system
SONAR: The Study Of Diabetic Nephropathy With AtRasentan
UACR: Urinary albumin-to-creatinine ratio
