# Supplementary material for: Insulin resistance, kidney outcomes and effects of the endothelin receptor antagonist atrasentan in patients with type 2 diabetes and chronic kidney disease
Source: Cardiovasc Diabetol. 2023 Sep 16;22:251. doi: 10.1186/s12933-023-01964-8 (PMC10505320; doi:10.1186/s12933-023-01964-8)
Supplement: Supplementary file 1 — Additional File 1: Table S1 with patient characteristics of the RADAR trial. [file 12933_2023_1964_MOESM1_ESM.docx]

**Supplementary table S1:** Patient characteristics of the RADAR trial

|  |  | **Atrasentan** | |
| --- | --- | --- | --- |
| **Characteristics** | **Placebo (n=30)** | **0.75mg/day** | **1.25mg/day** |
|  |  | **(n=59)** | **(n=64)** |
| Age, years | 63.0 (8.5) | 64.3 (9.6) | 64.0 (9.0) |
| Sex |  |  |  |
| Women | 8 (27%) | 10 (17%) | 17 (27%) |
| Men | 22 (73%) | 49 (83%) | 47 (73%) |
| Race |  |  |  |
| Asian | 4 (13%) | 6 (10%) | 9 (14%) |
| Black | 2 (7%) | 14 (24%) | 13 (20%) |
| Other | 1 (3%) | 3 (5%) | 4 (6%) |
| White | 23 (77%) | 36 (61%) | 38 (59%) |
| Body weight (kg) | 94.8 (18.1) | 92.8 (21.5) | 92.7 (17.3) |
| Blood pressure (mmHg) | |  |  |
| Systolic | 137 (15) | 138 (14) | 136 (14) |
| Diastolic | 71 (10) | 74 (10) | 74 (9) |
| eGFR, ml/min 1.73m2 | 46.4 (13.3) | 46.0 (14.1) | 48.7 (12.2) |
| UACR, mg/g | 633 (406 -1295) | 853 (526-1682) | 817 (425-1398) |
| Haemoglobin, g/L | 12.3 (1.6) | 12.7 (1.3) | 12.8 (1.7) |
| CVD disease | 7 (23%) | 13 (22%) | 2 (3%) |
| Insulin use | 11 (37%) | 23 (39%) | 22 (34%) |
| Diuretic use | 28 (93%) | 51 (88%) | 48 (75%) |
| Statin use | 23 (77%) | 47 (80%) | 53 (83%) |

**Abbreviations:** eGFR = estimated glomerular filtration rate; UACR = urine albumin creatinine ratio;
**Note:** number of patients included in the current analysis from the RADAR trial: placebo: n=26 (87%), atrasentan 0.75 mg: n=47 (80%), atrasentan 0.75 mg: n=50 (78%).
