# Supplementary material for: Insulin resistance, kidney outcomes and effects of the endothelin receptor antagonist atrasentan in patients with type 2 diabetes and chronic kidney disease
Source: Cardiovasc Diabetol. 2023 Sep 16;22:251. doi: 10.1186/s12933-023-01964-8 (PMC10505320; doi:10.1186/s12933-023-01964-8)
Supplement: Supplementary file 2 — Additional File 2: Table S2 with baseline characteristics of SONAR trial population for long-term outcomes. [file 12933_2023_1964_MOESM2_ESM.docx]

**Supplementary table S2:** Baseline characteristics of SONAR trial population for long-term outcomes

| **Characteristics** | **Included  (n=1102)** | **Excluded  (n=2566)** | **Total SONAR (n=3668)** |
| --- | --- | --- | --- |
| HOMA-IR | 5.9 [0.0-10.286.0] | NA* | 5.9 [0.0-10.286.0] |
| Age, years | 63.7 (8.8) | 64.8 (8.7) | 64.5 (8.8) |
| Sex |  |  |  |
| Women | 285 (25.9%) | 661 (25.8%) | 946 (25.8%) |
| Men | 817 (74.1%) | 1905 (74.2%) | 2722 (74.2%) |
| Race |  |  |  |
| Asian | 413 (37.5%) | 785 (30.6%) | 1198 (32.7%) |
| Black | 63 (5.7%) | 161 (6.3%) | 224 (6.1%) |
| Other | 51 (4.6%) | 85 (3.3%) | 136 (3.7%) |
| White | 575 (52.2%) | 1535 (59.8%) | 2110 (57.5%) |
| BMI | 30.2 (5.9) | 30.6 (5.5) | 30.5 (5.7) |
| Blood pressure (mmHg) |  |  |  |
| Systolic | 137.3 (15.7) | 137.1 (14.6) | 137.1 (14.9) |
| Diastolic | 75.9 (9.8) | 75.0 (9.7) | 75.3 (9.7) |
| eGFR, ml/min 1.73m2 | 41.7 (12.4) | 42.3 (12.9) | 42.1 (12.7) |
| UACR, mg/g | 861 [474-1674] | 817 [451-1517] | 829 [458-1556] |
| Haemoglobin, g/L | 128.6 (17.4) | 129.5 (17) | 129.3 (17.1) |
| BNP, pg/mL | 47 [25-88] | 48 [26-86] | 48 [26-87] |
| Hematocrit, L/L | 0.39 (0.05) | 0.39 (0.05) | 0.39 (0.05) |
| CVD history | 232 (21.1%) | 323 (12.6%) | 555 (15.1%) |
| Insulin use | 700 (63.5%) | 1615 (62.9%) | 2315 (63.1%) |
| Diuretic use | 881 (79.9%) | 2084 (81.2%) | 2965 (80.8%) |
| Statin use | 872 (79.1%) | 2041 (79.5%) | 2913 (79.4%) |

**Abbreviations:** HOMA-IR = Homeostatic Model Assessment for Insulin Resistance; BMI = body mass index; eGFR = estimated glomerular filtration rate; UACR = urine albumin creatinine ratio; BNP = brain natriuretic peptide; CVD = cardiovascular disease
**Note:** *baseline HOMA-IR not available for patients excluded from this analysis; UACR, BNP and HOMA-IR: median (interquartile range); all other numerical values: mean (standard deviation);
