# Supplementary material for: Insulin resistance, kidney outcomes and effects of the endothelin receptor antagonist atrasentan in patients with type 2 diabetes and chronic kidney disease
Source: Cardiovasc Diabetol. 2023 Sep 16;22:251. doi: 10.1186/s12933-023-01964-8 (PMC10505320; doi:10.1186/s12933-023-01964-8)
Supplement: Supplementary file 3 — Additional File 3: Table S3 with Association between HOMA-IR and long-term cardio-renal outcomes in participants not using insulin. [file 12933_2023_1964_MOESM3_ESM.docx]

**Supplementary table S3:** Association between HOMA-IR and long-term cardio-renal outcomes in participants not using insulin.

| **Outcome** |  | **Model 1** |  | **Model 2** |  | **Model 3** |  |
| --- | --- | --- | --- | --- | --- | --- | --- |
|  | **n/N events (%)** | **HR (95% CI)** | ***p* value** | **HR (95% CI)** | ***p* value** | **HR (95% CI)** | ***p* value** |
| **Cardiorenal outcomes** | | | | | | | |
| Low HOMA-IR | 15/201 (8.1%) | (reference) |  | (reference) |  | (reference) |  |
| High HOMA-IR | 23/201 (12.9%) | 1.43 (0.74-2.76) | 0.283 | 1.71 (0.83-3.51) | 0.146 | 1.99 (0.95-4.15) | 0.067 |
| per log unit increase | *NA* | 1.07 (0.77-1.49) | 0.685 | 1.20 (0.85-1.70) | 0.306 | 1.30 (0.91-1.86) | 0.145 |
| **Renal composite or all-cause mortality** | | | | | | | |
| Low HOMA-IR | 11/201 (5.8%) | (reference) |  | (reference) |  | (reference) |  |
| High HOMA-IR | 16/201 (8.6%) | 1.30 (0.60-2.83) | 0.511 | 1.50 (0.61-3.69) | 0.374 | 2.37 (0.95-5.95) | 0.066 |
| per log unit increase | *NA* | 1.00 (0.67-1.49) | 0.917 | 1.13 (0.73-1.75) | 0.585 | 1.44 (0.93-2.23) | 0.105 |
| **Renal composite** | | | | | | | |
| Low HOMA-IR | 8/201 (4.1%) | (reference) |  | (reference) |  | (reference) |  |
| High HOMA-IR | 14/201 (7.5%) | 1.49 (0.62-3.59) | 0.376 | 2.06 (0.69-6.10) | 0.193 | 3.89 (1.23-12.27) | **0.020** |
| per log unit increase | *NA* | 0.99 (0.62-1.56) | 0.964 | 1.15 (0.69-1.92) | 0.585 | 1.63 (0.94-2.85) | 0.084 |
| **CV composite** | | | | | | | |
| Low HOMA-IR | 8/201 (4.1%) | (reference) |  | (reference) |  | (reference) |  |
| High HOMA-IR | 9/201 (4.7%) | 1.09 (0.42-2.85) | 0.855 | 1.43 (0.51-3.98) | 0.492 | 1.33 (0.47-3.77) | 0.594 |
| per log unit increase | *NA* | 1.05 (0.65-1.71) | 0.834 | 1.28 (0.77-2.13) | 0.338 | 1.23 (0.73-2.06) | 0.440 |

**Note:**Model 1 covariates: age, sex, treatment, HOMA-IR
Model 2 covariates: age, sex, treatment, HOMA-IR, BMI, eGFR, log(UACR), SBP, DBP
Model 3 covariates: age, sex, treatment, HOMA-IR, BMI, eGFR, log(UACR), SBP, DBP, hemoglobin, cardiovascular disease history, log(BNP)
* in contrast to the analysis presented in table 2, there was insufficient data to include race as a covariate for this analysis.
