# Supplementary material for: Insulin resistance, kidney outcomes and effects of the endothelin receptor antagonist atrasentan in patients with type 2 diabetes and chronic kidney disease
Source: Cardiovasc Diabetol. 2023 Sep 16;22:251. doi: 10.1186/s12933-023-01964-8 (PMC10505320; doi:10.1186/s12933-023-01964-8)

**Supplementary figure 1:** Geometric mean HbA1c (95%CI) during the SONAR trial for patients with a HbA1c measurement at randomization.


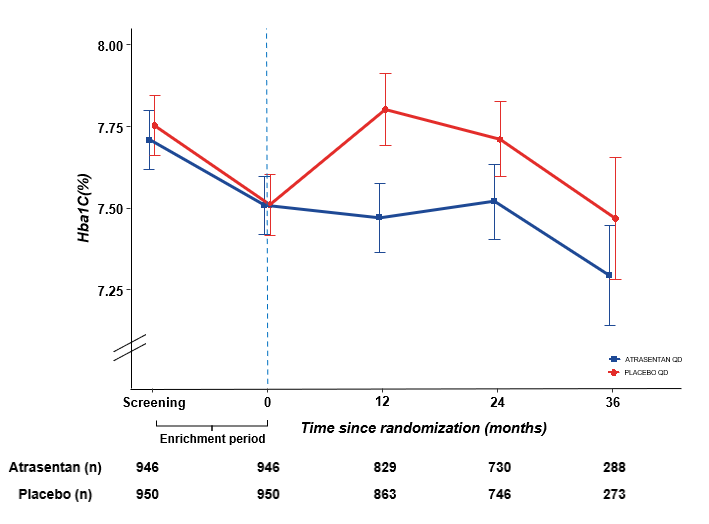

Supplement: Supplementary file 4 — Additional File 4: Figure S1 with HbA1c during the SONAR trial for patients with a HbA1c measurement at randomization. [file 12933_2023_1964_MOESM4_ESM.docx]
